# The default modes of reading: modulation of posterior cingulate and medial prefrontal cortex connectivity associated with comprehension and task focus while reading

**DOI:** 10.3389/fnhum.2013.00734

**Published:** 2013-11-12

**Authors:** Jonathan Smallwood, Krzysztof J. Gorgolewski, Johannes Golchert, Florence J. M. Ruby, Haakon Engen, Benjamin Baird, Melaina T. Vinski, Jonathan W. Schooler, Daniel S. Margulies

**Affiliations:** ^1^Department of Psychology, University of YorkHesslington, North Yorkshire, UK; ^2^Max Planck Research Group: Neuroanatomy & Connectivity, Max Planck Institute for Human Cognitive and Brain SciencesLeipzig, Germany; ^3^Department of Social Neuroscience, Max Planck Institute for Human Cognitive and Brain SciencesLeipzig, Germany; ^4^Department of Psychological and Brain Sciences, University of California Santa Barbara, CA, USA; ^5^Department of Psychology, Neuroscience and Behavior, McMaster University Hamilton, ON, Canada

**Keywords:** default mode network, reading, mind wandering, self-generated thought, comprehension, posterior cingulate cortex, medial prefrontal cortex (MPFC)

## Abstract

Reading is a fundamental human capacity and yet it can easily be derailed by the simple act of mind-wandering. A large-scale brain network, referred to as the default mode network (DMN), has been shown to be involved in both mind-wandering and reading, raising the question as to how the same neural system could be implicated in processes with both costs and benefits to narrative comprehension. Resting-state functional magnetic resonance imaging (rs-fMRI) was used to explore whether the intrinsic functional connectivity of the two key midline hubs of the DMN—the posterior cingulate cortex (PCC) and anterior medial prefrontal cortex (aMPFC)—was predictive of individual differences in reading comprehension and task focus recorded outside of the scanner. Worse comprehension was associated with greater functional connectivity between the PCC and a region of the ventral striatum. Better comprehension was associated with greater functional connectivity with a region of the right insula. By contrast reports of increasing task focus were associated with functional connectivity from the aMPFC to clusters in the PCC, the left parietal and temporal cortex, and the cerebellum. Our results suggest that the DMN has both costs (such as poor comprehension) and benefits to reading (such as an on-task focus) because its midline core can couple its activity with other regions to form distinct functional communities that allow seemingly opposing mental states to occur. This flexible coupling allows the DMN to participate in cognitive states that complement the act of reading as well as others that do not.

## Introduction

The development of writing has profoundly changed our social world: it allows ideas to be publicized to geographically diverse groups of individuals and permits concepts to be passed from generation to generation. More prosaically, but no less importantly, reading is a source of enjoyment available to every literate individual. Despite the value of reading, it can often seem that our minds are ill suited to the task of narrative comprehension. It is relatively common during reading, for example, to experience thoughts and feelings that are unrelated to the prose in front of us. Such *task-unrelated thinking* occurs across many task contexts (Smallwood and Schooler, 2006) and when it occurs in reading is a well-documented correlate of comprehension problems (Schooler et al., 2004; Smallwood et al., 2008; Franklin et al., 2011; McVay and Kane, 2011). At present we lack a detailed appreciation of the processes that govern how our thoughts are constrained to the narrative of a piece of prose, or those that cause our minds to wander away from what we are reading.

The current study used resting-state functional magnetic resonance imaging (rs-fMRI) to investigate whether variations in the experience of reading across individuals have a basis in the brain’s functional architecture. We were particularly interested in how the reading experience of different individuals varied with the behavior of the anterior medial prefrontal cortex (aMPFC) and posterior cingulate cortex (PCC), two major midline hubs of the default mode network (DMN; Raichle et al., 2001; Greicius and Menon, 2004; Buckner et al., 2008; Andrews-Hanna et al., 2010). The DMN is a constellation of brain regions including regions of prefrontal, parietal and temporal cortex that were initially discovered to “deactivate” during externally driven tasks, and also exhibit correlated intrinsic neural activity. The DMN consists of a *midline core* that includes the aMPFC and the PCC that can flexibly couple its activity to two additional subsystems: (i) a medial temporal lobe (MTL) subsystem that includes the hippocampus, the medial orbitofrontal cortex, and the temporal poles, and is important in episodic memory; and (ii) a *dorsal* subsystem that includes a dorsal region of the medial prefrontal cortex and lateral regions of the parietal cortex including the temporoparietal junction, and may be important in simulating of self and other (Buckner et al., 2008; Andrews-Hanna et al., 2010).

Currently the functions of the DMN are a matter of debate: prior studies have documented that the DMN plays a role in task-unrelated thought in sustained attention tasks (Mason et al., 2007; Christoff et al., 2009; Stawarczyk et al., 2011) as well as absent-minded lapses (Eichele et al., 2008; Christoff et al., 2009), suggesting that activity in this network could be responsible for the comprehension deficits that occur when the mind wanders during reading. Given that the DMN is implicated in task-unrelated thinking, it would seem intuitive that this network would be responsible for failures in comprehension that accrue due to mind-wandering.

Despite the appeal of the account of the DMN as the substrate for task-unrelated thought, the picture is likely to be more complicated: regions of the DMN have also been implicated in processes that are likely to be engaged during reading. For example, Fletcher et al. (1995) demonstrated that reading stories containing a narrative relating to either physical reality or to the mental states of other individuals activated the PCC relative to a series of unlinked sentences. Additionally, grey matter volume in the PCC is correlated with an individual’s capacity for phonological decoding (He et al., 2013). Recent meta-analyses have confirmed the role of the PCC and aMPFC in extended narrative comprehension, especially of a fictional nature (Mar, 2011).

Functional studies suggest the DMN is also implicated in processes that readers could use when reading. For example, the DMN is activated when participants make mental state attributions (Spreng et al., 2009; Spreng and Mar, 2012), when they retrieve information from memory (Huijbers et al., 2011), and has been hypothesized to allow for multiple different mental states all of which rely on information from memory to guide behavior (Smallwood et al., 2013). Meta-analyses also indicate that elements of the DMN are important in semantic processing: a capacity which is critical for reading (Binder and Desai, 2011). Semantic processing, mental state attribution, and the retrieval of information from memory are all abilities that are important in narrative comprehension because they allow the reader to understand the motives of characters and to make links between different elements of an extended text.

To understand why the DMN could be implicated in both costs and benefits to reading (Smallwood, 2013), we recorded how effectively individuals stayed on task while they read three different expository texts from an engaging popular work describing the history of science as well as the lives of the protagonists who contributed to this story. Afterwards participants completed a set of open-ended questions assessing their comprehension of what they read. Previously recorded rs-fMRI data was also available from a subset of these individuals which we used to explore how trait differences in the effectiveness of reading, measured both subjectively and objectively, were related to differences in the intrinsic functional connectivity of two of the major hubs of the DMN (aMPFC and PCC).

Based on prior evidence of DMN activation associated both with processes engaged by reading and with mind-wandering, as well as evidence that mind-wandering during reading is associated with poorer reading performance, we predicted that differences in reading effectiveness would relate to distinct patterns of functional connectivity between the midline core of the DMN and other brain regions. Prior studies have looked at how the behavior of resting state networks associated with reading behavior (Koyama et al., 2011), here we report an rs-fMRI investigation that is focused on the DMN. We used hypothesis driven seed-based analysis to examine whether metrics of reading behavior modulate the whole-brain connectivity of these regions. We were particularly interested in whether any observed modulations of functional connectivity could help shed light on how the DMN can be both beneficial and costly to the act of reading. Although this approach is limited by our choice of seed regions, it does not constrain the results of our analysis and so provides a straightforward, hypothesis driven method to assess our question of interest (Cole et al., 2010).

## Methods

Healthy participants were recruited for the current experiment from the Max Planck Institute for Human Cognitive and Brain Sciences participant database. Ethical approval was obtained prior to completing this experiment from the Ethics committee of the University of Leipzig (Ref # 360-10-13122010). Participants provided written informed consent prior to their participation.

### Assessment of reading behavior

Individual differences in maintaining focus on the narrative while reading and subsequent text comprehension was assessed in a sample of 61 healthy native German speakers (age range = 19–50, mean = 27.9 years, (SD = 5) 37 females, all right handed) while they read three excerpts from the official translation of Bill Bryson’s *A Short History of Everything,* a text that has been used in English language studies of mind-wandering (Smallwood et al., 2009b; Smilek et al., 2010). These texts are engaging non-fiction works that describes the characters and historical contexts of important scientific events. One text dealt with biology, a second with chemistry and a third dealt with geology. On average each text was approximately 1200 words long (mean = 1187). The Fleisch Kincaid levels were calculated using an online website^1^ and ranged from 34–39 making these texts slightly easier than university level material.

Participants read each text on a computer screen in an individual testing booth. Text was presented one sentence at a time and participants received experience sampling probes at random intervals (5–7 times for each text). Reading was self-paced and lasted approximately one hour in total. Experience sampling probes asked participants to report whether, at the moment prior to the probe, they were focused on what they were reading or on something else, a phenomenon described as task-unrelated thought (“Dachten Sie an die Aufgabe oder an etwas anderes?”). Participants answered this probe using a slider operated via a mouse. At the end of each text participants were asked about their familiarity with the text measured using a similar slider as was used to assess task focus.

After each text, comprehension of the material was assessed using a set of open-ended paper and pencil questions. On average participants took approximately 15 (±5) min to read each text and approximately the same time for answer the questions.

### MRI Acquisition and Analysis

rs-fMRI scans of 42 participants (age range = 19–26, mean = 27 years, 21 females) were retrieved from the existing Max Planck Institute for Human Cognitive and Brain Sciences participant database. The scans were collected on either a 3.0 Tesla Siemens Tim Trio (*n* = 26) or a Siemens Vario scanner (*n* = 16) before the reading assessment. Scanning duration varied from 360–600 s and two different slice order acquisitions were employed (ascending, *n* = 38, interleaved, *n* = 4). For all participants, the remaining parameters were the same (Repetition Times (TR) = 2000 ms; Echo Time (TE) = 30 ms; flip angle = 90°; s acquisition matrix = 64 × 64; Field of view (FOV) = 192 mm; acquisition voxel size = 3 × 3 × 4 mm). Participants were instructed to relax, to hold as still as possible, and to keep their eyes open. High-resolution *T1*-weighted anatomical scans were also acquired for all participants (MPRAGE, TR = 2300 ms; TE = 2.96 ms; TI = 900 ms; flip angle = 9°; FOV = 256 mm; acquisition voxel size = 1 × 1 × 1 mm).

### Functional Connectivity Analysis

#### Preprocessing, seed selection, and time series extraction

Cortical surface reconstruction was performed on the T1 scans using FreeSurfer (Dale et al., 1999; Fischl et al., 2001, 2002, 2004; Behzadi et al., 2007; Reuter et al., 2010). For each subject, nonlinear transformation from T1 to MNI template (created from 152 subjects, resampled at 2 mm, provided with FSL) was calculated using Advanced Neuroimaging Tools (ANTs; Avants et al., 2011).

To remove potential scanner instability effects the first four volumes of each echo planar imaging (EPI) sequence were removed. This was followed by simultaneous slice-timing and motion correction using 4DRealign implemented in nipy^2^ (Roche, 2011). Affine transformation from mean EPI image to T1 volume was calculated using BB Register (Greve and Fischl, 2009). Brain mask, cerebrospinal fluid (CSF) mask and white matter (WM) mask were extracted from FreeSurfer parcellation and transformed into EPI space (thresholded at 0.5 after interpolation). Realigned timeseries were masked using the brain mask. Principal components of physiological noise were estimated using the CompCor (Behzadi et al., 2007). Joined WM and CSF masks and voxels of highest variance were used to extract two sets of principal components (a.k.a. aCompCor and tCompCor). Using both of those strategies ensures robust estimation of physiological noise. Outliers in the EPI sequence were discovered based on intensity and motion parameters (ArtDetect).^3^ This was followed by denoising of the time-series using a general linear model (GLM) model with motion parameters, CompCorr components, and outliers as regressors (note that global signal was not regressed). Time-series were also smoothed using SUSAN with 5 mm full width half minimum (FWHM) kernel (Smith, 1992). Finally high pass (0.1 Hz) and low pass (0.01 Hz) filters were applied using FSL. Quality of scans and preprocessing was assessed visually by looking at EPI to T1 coregistration overlay, motion parameters plots and temporal signal to noise ratio volumes (tSNR).

To estimate connectivity from aMPFC and PCC, we selected spherical regions of interest (ROIs) of 6 mm radius with centers at MNI (x, y, z) from left aMPFC = −6, 52, −2, right aMPFC = 6, 52, −2, left PCC = −8, −56, 26, and right PCC = 8, −56, 26. These correspond to the major hubs of the DMN in the left hemisphere as reported by Andrews-Hanna et al. (2010) and we transformed them to the right hemisphere to reflect the fact that the DMN has a complex bilateral structure in normal healthy controls (Swanson et al., 2011). ROI masks were transformed back to each subject’s EPI space using combined inverse nonlinear MNI to T1 transform and affine T1 to EPI (thresholded after interpolation at 0.5). Translated ROIs were restricted within the brain mask. ROIs time-series were estimated by averaging voxels within each ROI. Full brain connectivity (correlation) maps were calculated using analysis of functional neuroimages (AFNI). Connectivity maps were Fisher’s r-to-z transformed and spatially transformed to MNI space for group-level analysis. Finally, these maps were averaged to provide an average connectivity maps for both regions across hemispheres. These averaged maps were used in the subsequent analysis.

Preprocessing was performed with a workflow from Brain Imaging Pipelines^4^ and all data processing integrated using Nipype (Gorgolewski et al., 2011). Code used for generating connectivity maps is available at https://github.com/NeuroanatomyAndConnectivity/pipelines/tree/reading_by_default/src/reading_by_default. Unthresholded statistical maps have been uploaded to NeuroVault.org and are available at http://neurovault.org/collections/59.

#### Analysis strategy

The answers to comprehension questions were scored according to an agreed rating system by two independent raters who were blind to the other’s rating and showed high agreement (> 90%). The resulting average scores for each question showed reasonable inter-item agreement (*Cronbachs Alpha* = 0.78). For each participant, an overall comprehension score was computed by averaging the scores obtained across all questions.

For the behavioral analysis we used a linear mixed model (LMM) as implemented in statistical package for the social sciences (SPSS) to examine how the subjective experience while reading was related to the objective measure of comprehension. Building on prior research detailing that poor comprehension can be a marker for task unrelated thought, we predicted the subjective reports of participants based on their comprehension of what they were reading. LMMs allow the comparison of predictors recorded on multiple occasions within different participants in a single model and so allowed us to assess whether variation in task focus within an individual and between different texts is associated with changes in comprehension level. In this analysis the participant was treated as a random effect, the task focus score was the dependent variable and the comprehension of what was read was included as a continuous independent factor. We also included age and gender as fixed factors in this model. We recruited a larger number of subjects for the behavioral element of the study to ensure that the relationships described were as robust as possible.

For the rs-fMRI analysis, SPM8 was used to perform a second-level random effects analysis of the patterns of connectivity from each ROI. This model included two between-participant regressors for each seed region: (i) reports of subjective focus during reading and (ii) average comprehension of the information read in the text. Scan length was also included in the model as a nuisance regressor. To perform inference we used the topological False Discovery Rate (FDR) approach (Chumbley et al, 2010): (1) statistical maps were thresholded at *p* < 0.01 (uncorrected) to define clusters; (2) the probability of the size of each cluster (given the search space, smoothness, and cluster forming threshold) was calculated using Random Field Theory (Worsley et al., 1992); (3) Benjamini-Hochberg procedure was applied to these *p*-values to maintain FDR (of clusters) at 5% level (Chumbley et al, 2010). These maps reflect the relationship between subjective and objective measures of reading behavior and the modulations of the intrinsic functional architecture of two key seeds of the DMN. In order to create scatter plots, the beta weights of the peak voxel from the cluster identified by the group-level analysis were calculated for each individual using SPM. To account for the different scanning sequences that our participants had we included three nuisance regressors: scanner type (1 or 0), scanning length in volumes (range 180–300), and slice-timing order scheme (1 or 0).

## Results

### Behavioral results

**Figure 1:**
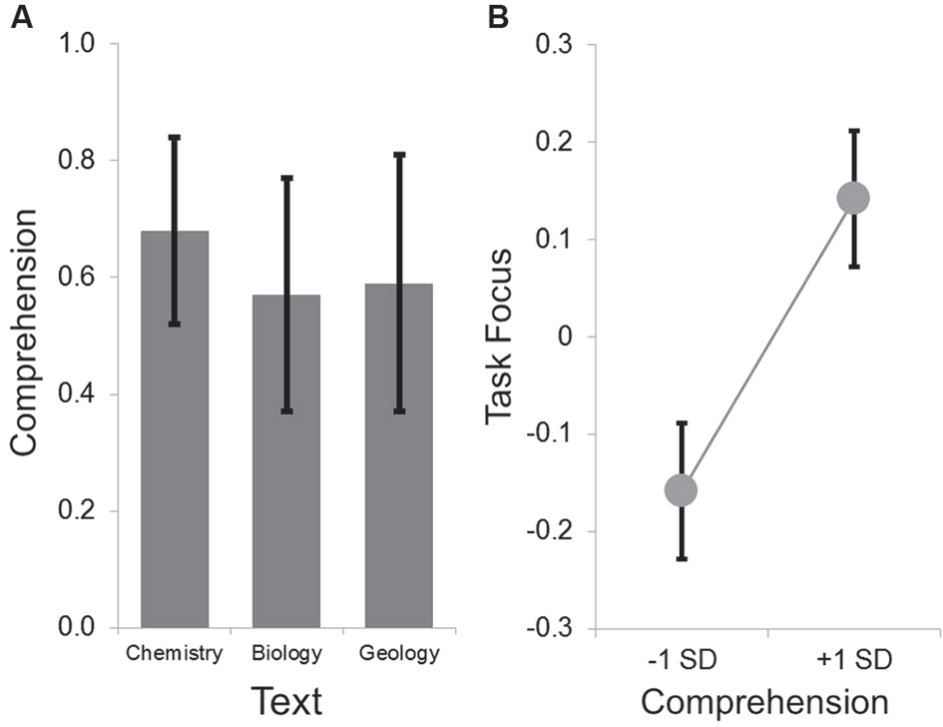
**Group level behavioral results**. The group average for the comprehension ranged from .5 to .7 across the three texts, indicating that the measure was not a floor nor at ceiling. This is summarized in Panel **(A)**. LMMs comparing the within participant variation in comprehension and task focus indicated that on texts when comprehension was higher, participants reported better task focus. This is summarized in Panel **(B)** in which the parameter estimates extracted from the LMMs were used to estimate the task focus at one standard deviation above and below the mean. In both panels the error bars indicate the standard error of the mean.

Comprehension was acceptable for each text (see Figure 1). Prior to analysis, all variables were *z*-transformed. The LMM revealed the expected positive relationship between higher ratings of task related focus when participants read a text when their subsequent comprehension was higher (*F*(1, 171.83) = 4.37, *p* < 0.05, *t*(171.83) = 2.09). This indicates that within participant changes in task focus while reading can be predicted based on whether a participant was successfully able to answer comprehension questions on the material presented during the period when attention was sampled (Smallwood et al., 2008). Neither age nor gender were associated with differences in task focus (all *p*-values < 0.3). We repeated this analysis including a participant’s familiarity with the text, and this did not appreciably alter the main effect of comprehension on task focus (*F*(1, 137.59) = 3.89, *p* = 0.051, *t*(137.59) = 1.97), nor was there an effect of familiarity on ratings of task focus or an interaction with comprehension (for both comparisons *p*-values < 0.3).

For the purpose of the group-level fMRI analysis we calculated individual differences in comprehension for each individual, as well as in the maintenance of task focus on what was read. Boxplot analysis indicated that one individual was an outlier on the measure of task focus, and this score was manually replaced with a score that was adjacent to the next highest score. At the group-level, comprehension and task focus were uncorrelated (*r* = −0.06, *p* = 0.667).

### Functional imaging results

**Figure 2:**
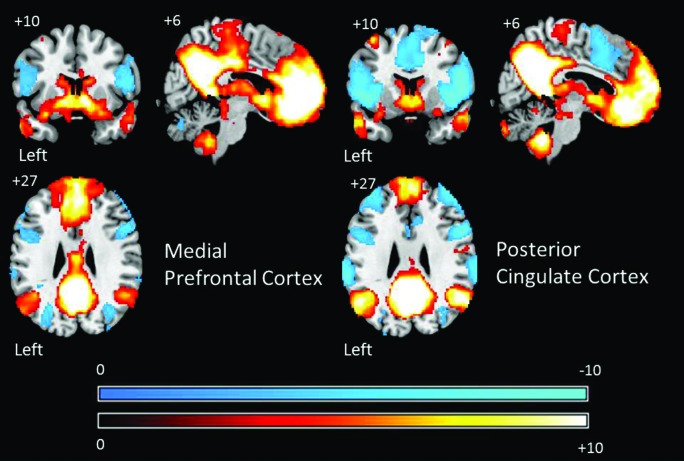
**Group level patterns of connectivity from bilateral seeds from the aMPFC and PCC employed in the current study**. These maps are presented to help visualize the group level patterns of connectivity upon which subsequent analysis are based. Warm colors indicate brain regions exhibiting positive correlations and cold colors indicate regions exhibiting negative correlations. For the purpose of display these maps are thresholded at a *T*-value of +/−3. All results are overlaid on a standard MNI template.

The connectivity patterns of the seed regions used in the current analysis are presented visually in Figure 2. These maps are thresholded with a *T*-value of +/−3 and provide a visualization of the patterns of connectivity upon which our subsequent analyses are based. Table 1 presents the results of the group level analyses.

**Table 1:** **Neural regions whose resting-state functional connectivity with the PCC was modulated by comprehension during reading and the aMPFC by task focus**.

**Seed**	**Relationship**	**k**	**Z**	**Peak Region**	**Peak Voxel**		
					**X**	**Y**	**Z**
aMPFC		392	4.74	Cerebellum	4	−58	−44
	On Task	762	4.62	Left Temporal Lobe	−40	−16	−16
		885	4.59	Left Parietal Cortex	−20	−54	42
		451	4.36	Cerebellum	10	−52	−10
		362	3.71	Posterior Cingulate	6	−74	38
PCC	Good Comprehension	424	3.62	Right Anterior Insula	52	22	10
	Poor Comprehension	371	4.02	Striatum	2	2	−12

Cluster forming threshold was set at p < 0.01 and significant clusters were determined using topological FDR (q-value < 0.05).

### Comprehension

**Figure 3:**
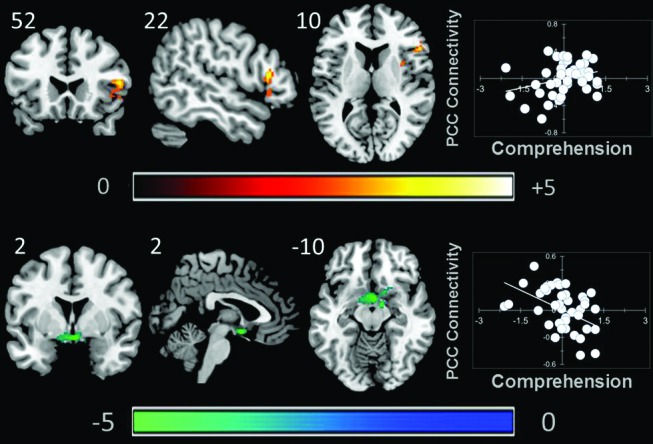
**Modulation of the connectivity pattern of the bilateral PCC by objective indicators of reading performance**. These images were created using a cluster forming threshold of *p* < 0.01. Topological FDR (*q*-value < 0.05) was used to correct for multiple comparisons. The scatter plots reflect the average correlation with the PCC for the peak voxel in each cluster. Each point is a single individual. Co-ordinates reflect the peak voxels for each cluster. All results are overlaid on a standard MNI template. Unthresholded statistical maps have been uploaded to NeuroVault.org and are available at http://neurovault.org/collections/59.

A whole brain search of the connectivity of PCC indicated that it was modulated by comprehension. Decreasing comprehension was associated with greater connectivity with a cluster between the PCC and the ventral striatum / amygdala. This can be seen in Figure 3. By contrast, as comprehension increased across individuals within our sample we found greater connectivity between the PCC and the right anterior insula (AI). Connectivity with aMPFC did not vary significantly with either better or worse comprehension.

### Task Focus

**Figure 4:**
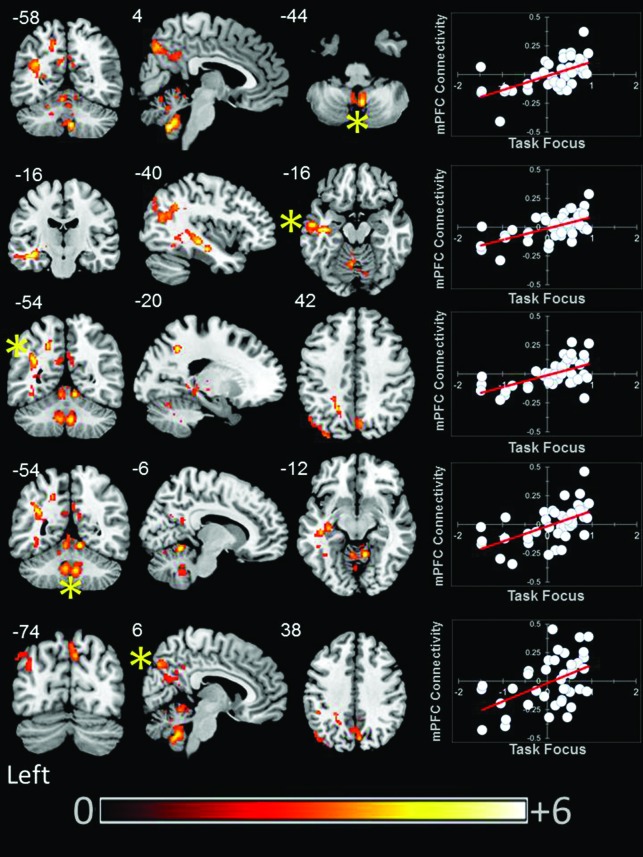
**Modulation of the connectivity pattern of the bilateral aMPFC by subjective indicators of reading performance**. These images were created using a cluster forming threshold of *p* < 0.01. Topological FDR (*q*-value < 0.05) was used to correct for multiple comparisons. The scatter plots reflect the average correlation with the aMPFC for the peak voxel in each cluster. Each point is a single individual. Co-ordinates reflect the peak voxels for each cluster. All results are overlaid on a standard MNI template. Unthresholded statistical maps have been uploaded to NeuroVault.org and are available at http://neurovault.org/collections/59/.

We found that aMPFC connectivity was modulated by better task focus. With increasing task focus, aMPFC exhibited greater connectivity with a region of precuneus/PCC, a cluster in the left parietal cortex, and a third cluster in the temporal cortex. aMPFC connectivity was also higher with two clusters within the cerebellum for individuals who stayed on task. These clusters are presented in Figure 4.

## Discussion

Our rs-fMRI examination suggests that variations in reading behavior are reflected by relatively complex associations with the connectivity patterns of both PCC and aMPFC. Although the objective (comprehension) and subjective (reports of task focus) indicators were uncorrelated at the group-level, our analysis using a LMM indicated that the measures were sensitive to within participant variance in the constructs measured. This suggests that fluctuations in task focus, and narrative comprehension showed a pattern of common variation within the individuals in our sample. At a group level we found that a more on task focus during reading was associated with greater functional connectivity between the aMPFC and the posterior cingulate and the precuneus, the left angular gyrus and temporal lobe, and the cerebellum. Although this result may seem contradictory with respect to the known role of the DMN in task-unrelated thought (e.g., Mason et al., 2007), this interpretation is consistent with evidence reviewed in the introduction that this network is involved in processes necessary for narrative comprehension, as well as more general evidence that integrity within the DMN is predictive of better rather than worse task performance (Kelly et al., 2008).

Objective variations in performance tracked with variations in connectivity of the PCC seed: as comprehension decreased across individuals we saw greater coupling between PCC and a cluster of subcortical regions including the inferior ventral striatum. By contrast, increasing comprehension was associated with greater coupling between the PCC and the right AI. Subjective descriptions of experience during reading varied with the connectivity of the aMPFC. As task focus increased across participants, the aMPFC showed greater coupling with a region of the posterior cingulate that overlapped with our seed region. This indicates that functional integrity within the midline core of the DMN is higher for people who stay focused during reading.

We also found evidence that suggests that an on task focus during reading is associated with greater coupling between the DMN and neural regions that support semantic processing. Individuals with higher on task reports exhibited greater connectivity between the aMPFC and a cluster in the left parietal cortex extending into the left angular gyrus and a second cluster that extended ventrally down the left temporal lobe and encompassed the hippocampus. The temporal lobe, especially on the left, is known to play an important role in semantic processes involved in language comprehension (Binder and Desai, 2011). Furthermore, grey matter volume is larger in a region of left parietal cortex for individuals who are especially competent at phonological decoding (He et al., 2013). In addition, the dorsal terminus of the cluster we identified in the parietal cortex (−45, −68, 26) is adjacent to the peak voxel of a region that has recently be found to be commonly activated by semantic tasks, as well as participating in the DMN (−48, −68, 28) (Seghier et al., 2010; Seghier and Price, 2012). If this region can participate in both semantic and DMN processes, it could afford the integration of processes supported by both systems. This could be one reason why we find greater connectivity between the aMPFC and this region with increasing on task reports across individuals: the ability to integrate semantic information with processes supported by the DMN would allow participants to build a richer model of what they are reading which would thus support an attentive focus on what is being read.

Comprehension was associated with differences in PCC functional connectivity. We found that better comprehension was associated with greater functional connectivity between the PCC and the right AI. Along with the anterior cingulate, these regions participate in what is known as the saliency network (Seeley et al., 2007). Our observation that individuals who have good comprehension shows heightened coupling between the right AI and the PCC is consistent with the notion that the saliency network plays an important role in coordinating the DMN (Menon and Uddin, 2010). For example, individuals with fronto-temporal dementia disease do not recruit the DMN when engaged in moral reasoning, a result that is thought to occur because of a diminished influence of the saliency network on DMN function (Chiong et al., 2013). Similarly, traumatic brain injury disrupts ongoing task performance in a go/no-go task due to a failure to deactivate the DMN, which occurs because of deficits in the integrity of WM tracts linking the hubs of the saliency network (anterior cingulate cortex (ACC) and AI). We hypothesize that the heightened coupling between the PCC and the right AI we observe could reflect a signature of an individual who can exert control over the processes served by the DMN which in turn would allow them to maximize their performance on tasks which require this network (such as reading). Interestingly, the right AI has been identified as the entry point of information into the saliency network, a finding which could explain why we find evidence in its connectivity with the PCC (Ham et al., 2013).

By contrast, worse comprehension was associated with greater coupling between the PCC and a region of the ventral striatum and this region is known to provide strong motivational signals that guide learning and behavior (Liljeholm and O’Doherty, 2012) and prior resting-state functional connectivity experiments have shown that this region of inferior ventral striatum shows a pattern of robust connectivity to PCC that more superior areas of the ventral striatum do not (Di Martino et al., 2008). Studies have found that activity in the ventral striatum exhibit reduced deactivation during cognitive tasks when individuals with attention deficit hyperactivity disorder (ADHD) are off medication, which is argued to result from motivational problems (Peterson et al., 2009). Individuals with ADHD also tend to show greater educational problems (Kuriyan et al., 2013), as well as greater mind-wandering (Shaw and Giambra, 1993). Recent studies have also found increased connectivity between the striatum and the PCC in adolescents with major depression (Gabbay et al., 2013), an observation that is relevant because greater mind-wandering and worse performance on objective measures of performance are correlates of states of dysphoria (Smallwood et al., 2007b), negative affect (Smallwood et al., 2009a) as well as clinical depression (Watts et al., 1988). Taken together we hypothesize that the pattern of heightened connectivity between PCC and striatum may be a neural signature that signifies problems in external task performance, perhaps due to motivational difficulties in assigning value to an external task (see also Mason et al., 2013). Support for this motivational account comes from experiments that show that performance-related financial reward improves performance and does so by reducing mind-wandering (Mrazek et al., 2012).

Our study found that the intrinsic functional connectivity of the two midline hubs of the DMN, and in particular the PCC, was such that they were capable of producing contrasting cognitive states: our results implicated the PCC in the production of distinctive, often opposing cognitive states (e.g., better and worse comprehension) through its cooperation with spatially distinct regions of the brain. The PCC is part of a highly corrected *rich club* of core nodes (Zuo et al., 2012; Sporns, 2013) that are more densely interconnected than would be expected by chance (van den Heuvel and Sporns, 2011) and many of the short communication paths within the brain go through one or more of its members (Harriger et al., 2012). These anatomical features would allow a neural region to control the dissemination of a large amount of neural communication (Sporns, 2013) and so would explain the PCC’s participation in different neural communities serving oppossing cognitive functions. It is noteworthy that we found that the the PCC either showed greater functional connectivity with the AI or the caudate/putamen depending on the level of a participants reading comprehension: all three of these regions are members of the rich club (van den Heuvel and Sporns, 2011).

In practical terms we demonstrated that rs-fMRI can be used to gain a functional description of the brain organization associated with reading behavior and it could be possible to use this approach in the future to understand attentional problems related to mind-wandering during reading (Smallwood et al., 2007a). For example, changing patterns of connectivity between regions of the DMN could provide an additional outcome measure for evaluating interventions that target improving educational achievement in individuals with difficulties in reading. Ultimately, given its sensitivity to individual differences in reading comprehension, rs-fMRI could even provide a method for the diagnosis of the specific functional problems that individuals have with reading problems.

Although our results are encouraging, the relatively small sample size (Yarkoni, 2009) means that our results must be treated with caution until they are replicated with a larger number of participants. Also, it should be noted that the LMM indicated the expected relationship between objective and subjective indicators of reading comprehension within participants. However, our rs-fMRI analysis is only focused on between participant differences. This prevents us from clearly dissociating the trait of mind-wandering while reading from the state of mind that occurs when our thoughts drift from what we were reading to task-unrelated concerns. To address this issue it would be necessary to acquire fMRI data with while reading is taking place, or at least using multiple sessions of both reading and rs-fMRI data on the same day. Also, it is important to note that we conducted a targeted investigation of the two key hubs of the DMN, and so our study does not provide any information on the behavior of other regions of the DMN or of the resting-state more generally.

In terms of the implications of our results for understanding the neural basis of reading, we suggest caution when considering the generality of our results. Given the complexity of tasks such as narrative comprehension, we expect that there are likely important boundary conditions on how the DMN contributes to this behavior. For example, it has been shown that this network exhibits greater negative correlation with the left fusiform gyrus for adults who read effectively, as assessed by capacities such as phonological expertise, whereas this relationship is reversed in children (Koyama et al., 2011). These results suggest that experience or age may influence the links between resting-state dynamics and reading abilities. Other studies have shown that patterns of connectivity predicting reading performance occur at multiple spatial scales (Wang et al., 2013), suggesting that the sub-components of large scale networks (such as the DMN) may play different roles during different phases of reading. It is also important to bear in mind that the neural processes engaged during reading can vary dependent on the texts factual or fictional composition (Altmann et al., 2012). Altogether such evidence suggests that for maximum relevance to educational research it would be valuable for future studies to examine the functional architecture that supports differences in reading experience for texts that vary on their narrative characteristics, as well as investigating how dynamics vary with the expertise of the reader. Both of these questions may also benefit from collecting neural data when participants are engaged in the act of reading. While these limitations provide an important caveat on our results, our data is nonetheless an important step in understanding the links between subjective experience and objective indicators of reading experience and their related neural correlates.

Finally, these data provide a plausible answer to the question that motivated this research in the first place: *Why should the DMN be implicated in both costs and benefits to reading?* We found that the mutual connectivity of the midline core of the DMN was such that it produced contrasting cognitive states. The aMPFC exhibited increasing coupling to a region of the PCC (encompassing the seed regions we used in our analysis) more for participants who reported maintaining focus on what they read. The PCC seed was more functionally coupled to the striatum for individuals who performed poorly on tests of comprehension, and more coupled to the AI for those who did well. Based on these data we can rule out simple accounts of the DMN as supporting either task-unrelated thought or successful reading. Instead we propose a novel hypothesis for why narrative comprehension suffers when the mind wanders (Schooler et al., 2004; Smallwood et al., 2008; Franklin et al., 2011; McVay and Kane, 2011): mind-wandering interferes with reading comprehension because it engages regions of the DMN that are also important in making sense of what is being read. This competitive hypothesis assumes that there are common processes that underlie an individual’s capability to make sense of what they are reading, which are also engaged by task-unrelated thinking. Whether the DMN helps or hinders the act of reading, therefore, may depend on whether its midline core is coupled to systems that represent the words on the page, or to the self-generated experiences that occupy our thoughts when the mind wanders.

## Conflict of interest statement

The authors declare that the research was conducted in the absence of any commercial or financial relationships that could be construed as a potential conflict of interest.

## Footnotes

^1^http://www.leichtlesbar.ch/html/_ergebnis.html

^2^http://nipy.org/nipy

^3^http://www.nitrc.org/projects/artifact_detect/

^4^https://github.com/INCF/BrainImagingPipelines
